# Segmental absence of the intestinal musculature in the stomach of an adult found during endoscopic submucosal dissection

**DOI:** 10.1055/a-2073-5044

**Published:** 2023-05-04

**Authors:** Kohei Funasaka, Noriyuki Horiguchi, Ryoji Miyahara, Tomoyuki Shibata, Yoshiki Hirooka

**Affiliations:** Department of Gastroenterology and Hepatology, Fujita Health University, Aichi, Japan

Segmental absence of intestinal musculature (SAIM) is a rare cause of spontaneous perforation in neonates 1 and is quite rare in adults 2 . Since 2020, SAIM has been reported to cause perforation during endoscopic submucosal dissection (ESD) of the esophagus 3 4 5 ; however, SAIM has never been reported in the stomach in adults. Herein, we report a case of SAIM identified during gastric ESD.

An 83-year-old woman underwent gastric ESD for two lesions (20-mm 0-IIa + IIc and 10-mm 0-IIa) that were located on the greater curvature of the gastric antrum ( Fig. 1 a ). Both procedures were performed using a needle knife (KD-1 L-1; Olympus, Tokyo, Japan) and an IT knife-2 (KD-610 L; Olympus), and with the use of carbon dioxide insufflation. After an incision had been made around a quarter of the circumference on the distal left side, we noticed that the submucosal area moved roughly synchronously with the patient’s respiration ( Video 1 ). Yellow tissue was observed through a relatively transparent submucosal layer, and was suspected to be fatty tissue outside of the stomach. The submucosal layer was easily retracted into the internal lumen by endoscopic aspiration. From the above findings, SAIM was strongly suspected. We carefully dissected the shallow layer of the submucosa just beneath the mucosal layer. Finally, we completed the ESD without any complications ( Fig. 1 b ) and closed the SAIM completely using clips ( Fig. 1 c ). The pathological findings showed a well-differentiated adenocarcinoma, which was confined to the mucosa and without vascular invasion.

**Fig. 1:**
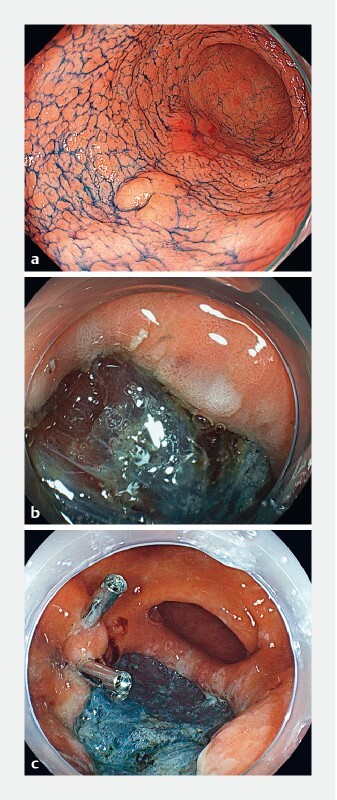
Endoscopic images showing: **a** the chromoendoscopic appearance of two neoplasms on the greater curvature of the gastric antrum; **b** the area where SAIM was suspected with an obvious depression after ESD; **c** the appearance after closure with clips.

**Video 1** Segmental absence of intestinal musculature is identified in the stomach of an adult during gastric endoscopic submucosal dissection.


A subsequent EUS was performed, 2 months after the ESD procedure, to assess the muscular propria beneath the scar ( Fig. 2 ). The fourth layer was found to be partially defective and overlapped discontinuously from each side ( Fig. 3 ). The findings of both the ESD procedure and the EUS strongly indicate the existence of SAIM.

**Fig. 2:**
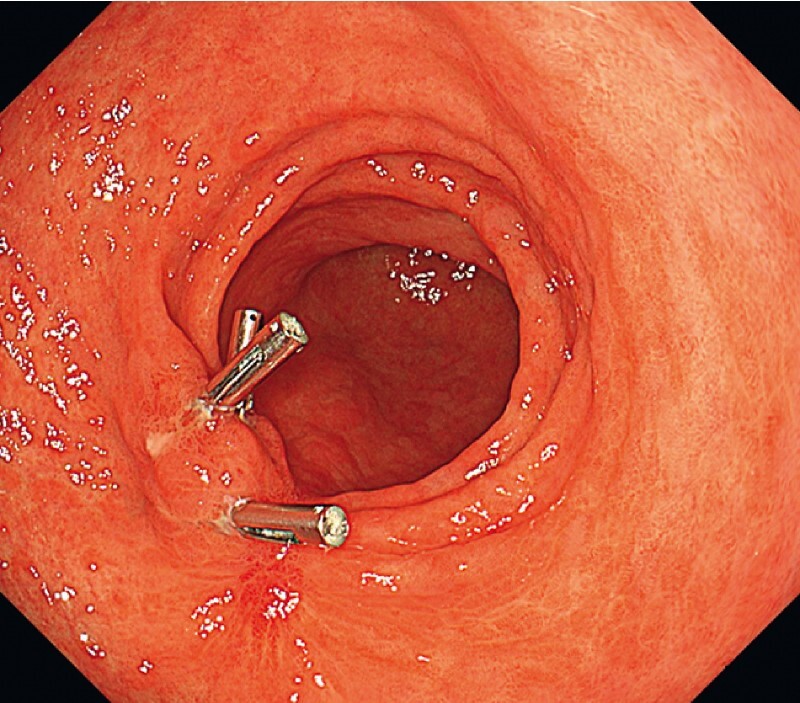
Image during a repeat endoscopy 2 months after the ESD showing the scar with clips remaining in place.

**Fig. 3:**
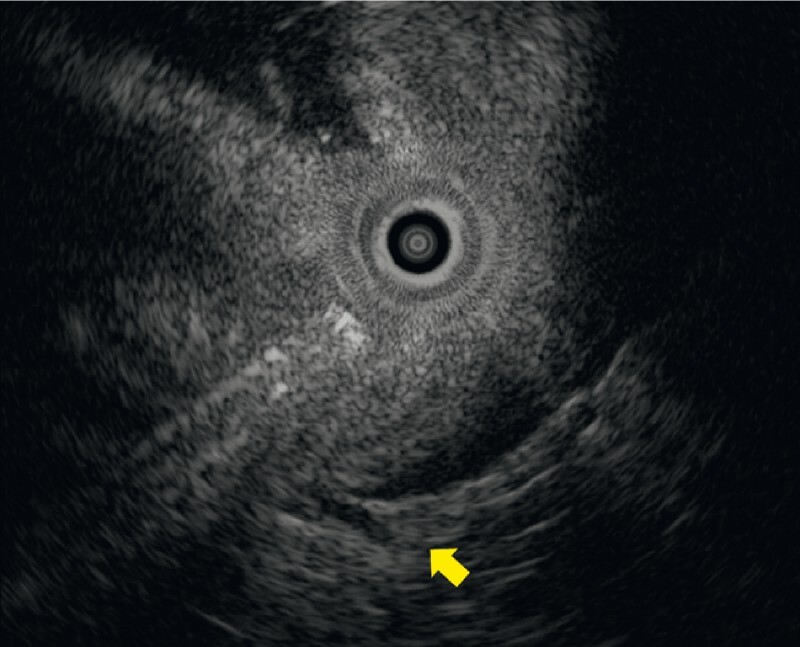
Endoscopic ultrasound image showing a partial defect of the fourth layer, which overlaps discontinuously from each side.

The existence of SAIM should be considered if the movement of the submucosal layer synchronizes with the patient’s breathing or if an unexpected perforation is observed during gastric ESD.

Endoscopy_UCTN_Code_CCL_1AB_2AD

## Notes

Endoscopy E-Videos

https://eref.thieme.de/e-videos
*E-Videos* is an open access online section of the journal *Endoscopy* , reporting on interesting cases and new techniques in gastroenterological endoscopy. All papers include a high-quality video and are published with a Creative Commons CC-BY license. Endoscopy E-Videos qualify for HINARI discounts and waivers and eligibility is automatically checked during the submission process. We grant 100% waivers to articles whose corresponding authors are based in Group A countries and 50% waivers to those who are based in Group B countries as classified by Research4Life (see: https://www.research4life.org/access/eligibility/ ). This section has its own submission website at https://mc.manuscriptcentral.com/e-videos
